# An intention to use mobile applications for medical supplies and equipment ordering in clinics

**DOI:** 10.3389/fpubh.2022.1021291

**Published:** 2022-10-20

**Authors:** Anas A. Salameh

**Affiliations:** Department of Management Information Systems, College of Business Administration, Prince Sattam Bin Abdulaziz University, Al-Kharj, Saudi Arabia

**Keywords:** emotion stage, fear effect, familiarity, mobile medical, MMSEOA, technology intention, reasons for, reasons against

## Abstract

This research developed a mobile medical supplies and equipment ordering app (MMSEOA) model and attempted to validate it empirically. When customers (clinic doctors) make purchases on the app, two types of reasons can be identified: “reasons for” include enduring involvement (emotions), product description, and awareness (familiarity) while the “reasons against”, were demonstrated as perceived risk and resistance to change (fear). This study aimed to strengthen and illuminate the most significant dimensions that enhance a doctor's understanding of MMSEOA and the intention to use it. Furthermore, this research investigated the model's applicability among clinic doctors in Jordan. The model was empirically examined using a sample of 342 Jordanian clinic doctors and their secretaries who use mobile services in general. The survey method, a quantitative approach, was utilized; the partial least squares structural equation modeling system was used to investigate the proposed framework. The results demonstrate that these “reasons for” positively influenced the intention to use the MMSEOA except product description. Similarly, reasons against negatively influence the customers' intention to use the MMSEOA app, while perceived risk had no effect on the intention to use. These findings suggested that researchers should focus more on the services, products, and the main function of the MMSEOA to determine their influences on customers' intention to use. This will improve the buying habits related to purchasing medical supplies using MMSEOA and other online platforms, specifically in Jordan and the Middle East at large.

## Introduction

Compared to traditional commerce, there is growing interest in mobile commerce services, also known as m-commerce. M-commerce has necessitated new approaches to advertising (1, 2). It affords every nation, business, and individual major business opportunities from a global perspective (3, 4). Furthermore, m-commerce facilitates the activities of small and medium enterprises (SMEs), which have also adopted m-commerce, similar to big enterprises, to develop their operations; m-commerce facilitates a strong relationship between retailers and end-users (5, 6).

M-commerce also plays a new role in the business world, affecting marketers and companies. It facilitates various business activities such as shopping, sports, advertisements and gaming (7). Variables related to the facilitating conditions, such as culture and/or telecommunications infrastructure, are also influenced by changing intentions to use m-commerce across countries (8). According to the Jordanian ICT Ministry, ~90% of Jordanian households have smartphones, 89% have Internet subscriptions, and 33% have computers (9). These indicators cannot provide enough understanding of m-commerce user attitudes. Therefore, to create suitable marketing tactics based on varying attitudes, it is necessary to comprehend the individual and environmental variables that determine the level of intention to use.

It can be assumed that all institutions, including businesses in Jordan, whether small or large and the government, have all the possible technological resources to effectively facilitate m-commerce (2). Jordan's strong telecommunication infrastructure enables businesses to connect to m-commerce services and adopt them for their activities. Furthermore, it can be observed that the number of services availed to smartphone users in Jordan by m-commerce is growing steadily. However, m-commerce will be worthless without mobile devices that can deal with digital data associated with digital communications (10).

Various drivers of the intention to use m-commerce services, such as perceived risk, compatibility, perceived usefulness, and perceived ease of use, have been identified in empirical studies (11). The extant literature examines the reasons for and barriers to technological acceptance by doctors and the adoption of mobile medical supplies and equipment ordering apps (MMSEOA) by different customers. Most prior research concentrated on examining the response to m-commerce adoption (12–14). In other words, earlier research has mostly considered the facilitators (reasons for) m-commerce adoption. Still, the equally essential barriers or inhibitors that create the customers' resistance toward using m-commerce have been rarely explored. However, a dearth of research examines both of these reasons within one framework (see Table 1). Claudy et al. (22) argued that researchers need to concentrate on identifying and examining the both acceptance and resistance factors for any invention or technology adoption behavior. According to social psychology research, these drivers of adoption and barriers factors are quantitatively different and influence the customers' consumption choices differently.

**Table 1:** Summary of prominent studies on m-commerce.

**Authors**	**Study context**	**Theoretical Support**	**Behavioral reasoning approach (BDA)**
Chau, Deng, and Tay (12)	Mobile commerce	Diffusion of innovation theory (DOI), Technology-organization-environment framework (TOE), Resource-based theory (RBT), Technology acceptance model (TAM)	Missing
Sim et al. (14)	Mobile commerce	Unified Theory of Acceptance and Use of Technology (UTAUT)	Missing
Salimon et al. (13)	Mobile commerce	Technology Acceptance Model 3 (TAM 3), Universal Theory of Acceptance and Use of Technology 2 and Technology-Organization-Environment	Missing
Vinerean, Budac, Baltador, and Dabija (15)	Mobile commerce	UTAUT 2	Missing
Sharma and Madan (16)	Mobile commerce	TAM	Missing
Abdallah et al. (17)	Mobile commerce	Extended TAM	Missing
Verma, Tripathi, and Singh (18)	Mobile commerce	Theory of planned behavior (TPB).	Missing
Pandey and Chawla (19)	Mobile commerce	UTAUT	Missing
Kargeti, Singh, Paul, and Sagar (20)	Mobile commerce	TAM	Missing
Ali, Khalid, Javed, and Islam (21)	Mobile commerce	Technology Readiness (TR)	Missing

This research bridges this gap and contributes to the literature by employing the behavioral reasoning approach (BRA) to understand customers' intentions to use MMSEOA. The research model was developed based on behavioral reasoning approach (BRA) and tested by conducting a cross-sectional survey of 342 Jordanian customers (i.e., medical doctors). BRA enables the researchers to determine the relative impact of “reasons for” (i.e., acceptance factors) and “reasons against” (resistance factors) on customers' intention toward using MMSEOA. BRA is an emerging approach that offers a complete understanding of the different behavioral components of customers' intentions to use MMSEOA (23). This study investigated MMSEOA and the clinic doctor's intention to use it. Studying the attitude of clinic doctors toward this service will grant marketers insight into the perceived related challenges, the intention to use and customer satisfaction (24). However, the gap aimed to be filled by this investigation is in the area of the usage of the MMSEOA and the reasons for (enduring involvement, product description and product awareness) and reasons against (perceived risk and resistance to change) affecting the clinic doctors' intention to use it. The MMSEOA provide multiple convenience to their users including access, search, evaluation, transaction, possession, and post-possession convenience. However, convenience develop positive attitude among users to use the apps such as MMSEOA and influence their intention to use it (25).

The present research is organized as follows: Section 2 includes a literature review, theoretical support, and hypotheses development. In Section 3, the research method has discussed. Section 4 demonstrates the analysis and result of the current study, and section 6 presents the study's discussion, implications, limitations, future directions and conclusions.

## Literature review and theoretical support

### M-commerce

For most people, m-commerce is a clear and concise concept. Some researchers consider it an extension and variation of e-commerce (26, 27). However, other scholars posit that m-commerce has deviated from the earlier concept of e-commerce (28). According to (29), the term m-commerce is arguably misleading because its value chain and business models are significantly different from e-commerce. Similarly, other researchers say m-commerce necessitates communication with users (30).

The key observable characteristics of m-commerce are dissemination, flexibility, personalization, and ubiquity. M-commerce is ubiquitous because it enables provisioners to reach their clients anytime, anywhere (31); in other words, users of m-commerce can obtain information wherever and whenever they want. This research differentiates between m-commerce and e-commerce. The researcher adopts the definition of m-commerce from Omonedo and Bocij (32), as “any deal, including the transfer of ownership or licenses to manage services and goods, which is started and/or executed by utilizing mobile access to computer-mediated systems by the guidance of mobile devices.” A summary of prominent studies on m-commerce is presented in Table 1.

### Behavioral reasoning approach

Previous studies have considered the customers' adoption of innovation by considering different theories, such as the theory of reasoned action (TRA), theory of planned behavior (TPB), technology acceptance model (TAM), Unified Theory of Acceptance and Use of Technology (UTAUT), Technology Readiness (TR) and diffusion of innovation theory (DOI). These theoretical models have been criticized since they have focused on the factor that affects acceptance while disregarding the variables associated with customer resistance (22, 23, 33). In addition, it is necessary to add the consumption barriers in any theoretical model because it enables the researchers to examine different cognitive factors that customers consider to formulate their intentions (34).

In addition, past research has demonstrated that new services and products have a high failure rate due to the lack of awareness of the numerous reasons related to consumer resistance or the barriers to their acceptance (35). In the context of m-commerce, the situation is the same as most previous research has centered on identifying the positive characteristics that influence mobile app usage intentions. However, there is limited knowledge about customers' willingness to use MMSEOA and policymakers are increasingly concerned that the problem should be investigated as quickly as feasible. Researchers argued that it is important to identify, formulate, and adopt novel behavioral models that might provide a more appropriate understanding of the elements that drive the acceptance and barriers to innovations (22).

BRA is a theoretical model that allows researchers and practitioners to examine the relative impact of “reasons for” and “reasons against” on customers' intentions toward using any innovation (23, 36). BRT differs from acceptance research models because the acceptance model considers only “reasons for” accepting new technology (37). Researchers have proposed that “reasons for” avoiding any innovation are not always the opposite of “reasons for” adopting the innovation (22, 23, 33). Therefore, a comprehensive understanding of consumer behavior requires an analysis of “reasons for” and “reasons against.” However, BRA not only enables researchers to differentiate between the “reasons for” and the “reasons against,” but it also facilitates analyzing the impact of these factors on customers' intents and behavior by considering a single research model (35).

### Conceptual framework

The researcher used the search engine “Google Scholar” to review the research articles on m-commerce by using various combinations of specific keywords, “m-commerce”, “mobile commerce”, and “intention to use” by “clinics”, “firms”, as well as “firms and gynecologists”. In addition, the “AND”, “OR”, and “^*^” operations were also used to identify the relationship of m-commerce with the “intention to use” it. Moreover, we established inclusion and exclusion criteria to ensure the relevancy and quality of articles considered in this systematic literature review (38). After analyzing the prominent and recent studies, researchers have found that there is no other study that has used the BRA to examine the customers' intention to use MMSEOA.

In addition, the reasons elicitation process was also performed to identify the “reasons for” and “reasons against” in the context of MMSEOA adoption. Before finalizing the reasons, the list of reasons was discussed with experts in the medical field, such as doctors from Jordan medical clinics having experience of using any form of technology to buy medical supplies and equipment. Semi-structured interviews (i.e., face-to-face) were conducted with ten doctors to determine both the “reasons for” and “reasons against” adopting MMSEOA. The methodology employed by Claudy et al. (22) and Westaby (36) was used to elicit the reasons. Respondents were asked to respond to a list of reasons why they intend to use MMSEOA. Respondents were asked to rate the likelihood of each of the following statements as the reason for adopting MMSEOA on a four-point scale ranging between 0 and 3 (i.e., “0” represents “not a reason”, “1” indicates “a somewhat influential reason,” “2” demonstrates “influential reason,” and “3” states “very influential reason.”) (36, 39).

**Figure 1:**
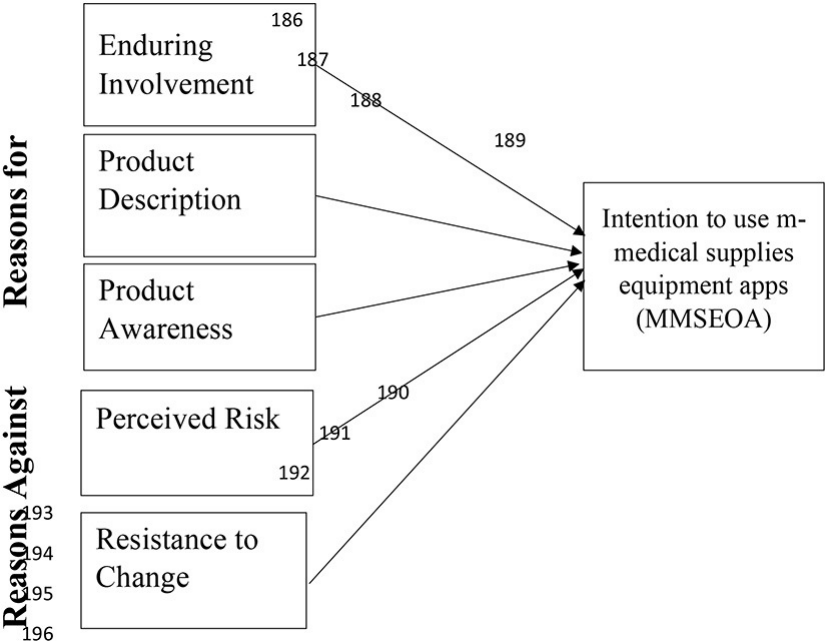
Research framework.

Consequently, based on the systematic literature review and semi-structured interviews, this research selects the top three “reasons for” using MMSEOA, including “enduring involvement (40, 41), product description (11, 42–44) and product awareness” (11, 45, 46) as prior research on MMSEOA has emphasized on these reasons. Similarly, the “two reasons against” namely, perceived risk (11, 47) and resistance to change (22) were selected that can affect the intention of consumers or clinic doctors, especially gynecologists, toward using m-commerce services. The relevant background on improving the intention to use m-commerce services and the development of the hypotheses of the study are presented. After reviewing the systematic literature and extracting reasons, five independent variables, including “reasons for” (enduring involvement, product description, and product awareness and “reasons against” (perceived risk and resistance to change), were identified as affecting the intention to use m-commerce as depicted in Figure 1. Further, the relationship between these independent variables and the dependent variable (i.e., intention to use MMSEOA) was examined.

### Intention to use m-commerce

Previous studies have shown that m-commerce delivers special benefits, including revenue streams, communication channels, markets, new product development, and new services, that do not exist in traditional e-commerce (27). Comparatively, m-commerce is user-friendly, and through hardware and its apparent commercial purposes, it has relative advantages over e-commerce. For instance, mobile devices are easier to use than PCs; further, in terms of cost and ubiquity, mobile devices have clear advantages over PCs, and the learning curve of mobile devices is more sustained compared to other technologies (48). The diversification and innovation in mobile technology have led businesses to invest more in improving their m-commerce involvement. Nowadays, people in developed countries, such as Jordan, exhibit a high intention to use mobile devices, and these changes positively impact how the devices are used. Therefore, the importance of the intention to use a mobile device can affect organizations and consumers.

Intention to use is a transaction attitude displayed by consumers after assessing services and goods (49). Based on a model presented by (50), the intention to use is the customer attitude determining whether the purchase will be made; a greater customers' intention to use indicates that a customer has a greater likelihood of buying the goods or services. Whenever customers plan to purchase any goods, they conduct their findings and collect information, based on practices and the environment, on the services or goods; after gathering sufficient information, they assess their interests and make decisions based on the information obtained (51).

Intention to use is an essential concept in marketing (52). In the context of m-commerce, it is the degree to which a customer intends to purchase online (53). According to Kotler (54), personal behaviors, attitudes, and varying factors impact customers' intention toward using the app. It is reinforced by price and advertisement (55), brand acknowledgment (56), and improved customer experience and knowledge of a brand (57, 58).

### Hypotheses development

#### Enduring involvement (emotion stage)-reasons for

Van den Berg et al. (59), Todorov et al. (60); Trumbo (61), through the theory of “attitude formation” and the “heuristic-systematic information processing model”, emphasized the fact that consumer involvement plays a vital part in mobile services. In addition, previous studies have found that consumers' behaviors and attitudes affect the success of a website (62–64).

Consumers require more time to evaluate the information that exists on websites. Further, more effort and time are expended on taking actions and making decisions (65, 66). Hollebeek, Jaeger, Brodie, and Balemi (67) stated that customers need to be more active to obtain more information on a platform, especially when they are highly involved in the process of making purchases. Thus, the more consumers are involved on a website, the more items are purchased on it (41, 68, 69).

Enduring involvement seems to positively influence the intention to use (40, 70) because consumers will make good purchase decisions when product information is available in detail. In addition, according to Jackson et al. (70), purchase experience on the platform steadily increases the enduring involvement and purchase intention. When the supplier is more involved, the customers will experience less uncertainties. Moreover, this will reduce the risks and costs that can occur during the purchasing process. Accordingly, consumers will spend more time on the platform, which, in the end, will increase the intention to use the platform. Based on the foregoing explanation, the following hypothesis was developed:

*Hypothesis 1 (H1). Enduring involvement is positively linked to the intention to use the MMSEOA*.

#### Familiarity with product- reasons for

##### Product description

Belanche et al. (71); Lu et al. (72); Srivastava and Chandra (43), considering the uncertainty reduction theory, posited that communicating with customers for a long time or deeply reduced customers' uncertainty and even enhanced the intention of purchasing. According to Pan and Chiou (73); Wells et al. (74), the availability of high-quality information on a product can strongly influence the understanding of the product to the customer and intention to purchase. It is important that consumers obtain high-quality details on products (73, 75, 76).

According to Walsh and Mitchell (44), quality information available on products across different social media enables customers to evaluate the characteristics of the product and decreases ambiguity; this increases the perceived trust toward the goods and the supplier, and by extension., the intention to use e-services. According to Landgrebe (77), the signal theory is useful in circumstances of ambiguity, such as the context of inadequate information. This theory can be further utilized to illustrate the nature of the relationship between trust and signals. Im and Ha (42) argued that customers might review the adequacy of the product information by searching for the information before the purchasing processes, which will affect their intention to purchase the product. Adequate amount of high-quality information leads customers to perceive the suppliers as responsible, to a particular extent, and reliable and trustworthy, which enhance the intention to use significantly. Consequently, the following hypothesis was proposed:

*Hypothesis 2a (H2a). Product description is positively linked to the intention of using MMSEOA*.

##### Product awareness

Many researchers have demonstrated that awareness significantly impacts the intention to use new technology. The intention to use is defined as “the strength of one's intention to perform a specified behavior” (78). According to (45) extensive knowledge of products and services may also impact the intention to use significantly. Marketers strive to create brand awareness using various marketing tools such as advertising and sales promotions. Brand loyalty, which eventually enhances the buying preference and customers' intention to use, is directly proportional to brand knowledge (46). Furthermore, a well-recognized brand will rouse a greater customers' intention to use than a less-recognized brand (79).

Similarly, Hoyer and Brown (80) described the effect of product awareness on choice, product sampling, and frequency and concluded that customers typically opted to buy or intended to buy products they were conscious about. According to GreenfieldOnline (81) an analysis of the responses of respondents to their questionnaire revealed that extensive description of products on the app significantly impacted the customer's intention to adopt it. Certainly, the intention to adopt m-commerce apps is similar to consumer awareness of the goods. Based on the foregoing, the following hypothesis was formulated:

*Hypothesis 2b (H2b). Product awareness is positively linked to the intention of using MMSEOA*.

#### Fear effect–reasons against

##### Perceived risk

The perceived risk is a major barrier to online payment for e-customers; therefore, majority of e-retailers and businesses consider and address issues related to online payment through security and technologies and measures such as awareness campaigns (82–84). It is not difficult to understand how electronic MMSEOA can be affected by perceived risk because a system failure is associated with information loss. Further, when the perceived risk is high, consumers are less likely to make purchases on online sites (85–87).

M-commerce naturally has factors that increase the perceived risk; for instance, it allows the use of the consumer's location to make location-based suggestions. According to Alliance (88); Slade et al. (47) the use of a consumer's financial data for payments on mobile devices and exploiting consumers' private data, such as social media, to perform services also increase the perceived risk (89, 90). A high level of perceived risk can negatively affect the intention to use different m-commerce services. According to Kondo and Ishida (91) the perceived risk significantly affected the intention to use mobile applications for entertainment or gaming (m-gaming). Similarly, Natarajan et al. (92) argued that the perceived risk had a notably negative adverse impact on m-shopping in India.

According to Udo et al. (93) generally, the perceived risk elicits bad or good feelings in e-customers, which is reflected in behavioral intention, attitudes, and beliefs. Thus, determining the effect of perceived risk on m-commerce is still being actively researched. Although according to a significant number of studies, the perceived risk negatively affects customer behavior toward online shopping, other studies have not found any impact at all. Following a review of all the relevant literature, it was concluded for the purpose of this study that a high level of perceived risk negatively influences the intention to use m-commerce services. Thus, the following hypothesis was developed:

*Hypothesis 3a (H3a). Perceived risk is negatively linked to the intention of using MMSEOA*.

#### Resistance to change

According to Chamberlain et al. (94); Dent and Goldberg (95), resistance behavior have various levels; resistance could manifest as malicious use, dysfunctionalities, and low levels of usage. From a practical perspective, consumer resistance can foster a broad diversity of behavioral patterns. The most popular theory on resistance is the theory of multilevel resistance (96), which identifies four levels of resistance: indifference, negative resistance, active resistance, and vigorous resistance. Sequentially, these four resistance levels can manifest as inaction, rejection, disappointment voicing, and defiance.

The presence of resistance, at any of these levels, is unacceptable on information systems. Thus, it is important to recognize the presence of resistance to change. As the use of the MMSEOA is totally optional, it may be hard to differentiate between apathy and resistance in doctors and/or clinics. A lack of awareness of the presence of MMSEOA services can produce inaction. Therefore, it is crucial to verify the existence or absence of resistance to change. According to Claudy et al. (22); Gurtner (97); Kleijnen et al. (98), consumer resistance has been identified as one of the primary challenges in the implementation of large-scale information systems. Lallmahomed (99) state that scant attention has been paid the concept of resistance to change, despite the fact that it is essential to study such inhibiting factors. Thus, the following hypothesis was formulated:

*Hypothesis 3b (H3b). Resistance to change is negatively linked to the intention of using MMSEOA*.

## Method

This practical analytical research investigated the effects of the reasons for (i.e., enduring involvement, product description and product awareness) and reason against (i.e., perceived risk and resistance to change) on the intention to use the MMSEOA. The research model, which was developed based on earlier studies and reasons elicitation, is presented in Figure 1.

### Sample and measures

In this research, items developed based on previous research were adapted and revised accordingly. The questionnaire surveyed the use of the MMESOA based on one component of the emotion dimension, enduring involvement, which is covered by five items. The two components of the fear effect, perceived risk and resistance to change, were covered by seven items. Product description and awareness, two components related to familiarity with products, were covered by six items. Finally, the intention to use (MMSEOA) was covered by three items. A five-point Likert scale was used; the responses ranged from 1 (“strongly disagree”) to 5 (“strongly agree”). The details of the items are listed in Table 2.

**Table 2:** Questionnaire items.

**Concepts**	**Variable**	**Item**
Platform emotion stage	Enduring involvement	Comfort while using the m-medical supplies and equipment ordering app is important.
		Comfort while using the m-medical supplies and equipment ordering app is of high interest.
		Comfort while using the m-medical supplies and equipment ordering app means a lot.
		Comfort while using the m-medical supplies and equipment ordering app is significant.
		Comfort while using the m-medical supplies and equipment ordering app matters a lot.
Fear effect	Perceived risk	I worry about credit card information being stolen when I use the m-medical supplies equipment ordering app.
		I worry about the m-medical supplies and equipment ordering app quality on the Internet.
		When using the m-medical supplies and equipment ordering app, I worry about transaction safety online.
		I worry when I use the m-medical supplies equipment ordering app about how my personal information might be used when I buy online.
	Resistance to change	I would not alter my decision to order by switching from using traditional pharmaceutical companies to using the m-medical supplies and equipment ordering app.
		I would not willingly alter my decision to order using traditional pharmaceutical companies to using the m-medical supplies and equipment ordering app.
		I would not switch from using the traditional pharmaceutical companies to using the m-medical supplies and equipment ordering app.
Familiarity with product	Product description	The m-medical supplies equipment ordering app explanations were clear.
		I was capable to understand the explanations obtained on the m-medical supplies equipment ordering app items.
		The m-medical supplies equipment ordering app descriptions were hard to understand.
	Product awareness	I am aware of the items on the m-medical supplies and equipment ordering app.
		I can recall the items on the m-medical supplies and equipment ordering app.
		I can recognize the items on the m-medical supplies and equipment ordering app.
Intention to use MMSEOA		I expect to buy goods on the m-medical supplies and equipment ordering app.
		I would buy goods on the m-medical supplies and equipment ordering app.
		I intend to purchase products on the m-medical supplies and equipment ordering app.

The survey used for the study was empirically performed from December 20, 2019, to March 10, 2020, on clinic doctors who were familiar with MMSEOA. The survey was administered using a convenience sampling method; clinics from different places in the Hashemite Kingdom of Jordan were selected. The researcher aimed to cover all the regions (north, center, and south). The questionnaires were distributed directly to the target respondents and retrieved. Of the 460 questionnaires distributed, 393 were returned; 37 were incomplete, and 14 of the respondents mentioned that they did not use the MMSEOA. Thus, 342 questionnaires were analyzed.

### Data analysis

The research model was examined using the partial least squares structural equation modeling (PLS-SEM) (100). The partial least squares regression was adopted for several reasons. The research model is somewhat complicated, based on the hypothesized type of relationships among the independent and dependent variables. In addition, the dimensionality of the higher-order constructs—(1st and 2nd) was examined. The current research utilized the latent scores of the variables within the consequent investigation of predictive relevance, especially toward implementing the approach of two-stage for forming the multidimensional variables.

Finally, in the current study, the principal dependent logical variables for character instance (MMSEOA) were considered to be determined. This means that this research depended on a framework with a reflective design approach in a composite measurement model. Further, the correlations among the independent and dependent variables were also considered. Hence, the traditional PLS suited the context of this research (101). For this research, the SmartPLS 3.2.8 software (102) was utilized.

## Results

### Participants and demographic information

In the current survey, there was a response percentage of 85.43% (393 questionnaires); therefore, 67 questionnaires, representing 14.56%, were not returned. Of the 393, it was observed that 37 questionnaires were not filled; thus, they were excluded. Moreover, 14 of the respondents stated that they did not utilize the MMSEOA. This meant that the responses of only 342 (74.34%) respondents were analyzed. The review of the respondents' demographic is shown in Table 3.

**Table 3:** Respondents' demographic information.

**Construct**	**Category**	**Count**	**Percentage**
How long have you been using m-medical supplies equipment ordering apps	<1 year	161	47.1
	Between 1 to 3 years	70	20.4
	Between 4 to 6 years	31	9.1
	More than 6 years	80	23.4
Total		342	100
List of URLs	https://www.hikma.com/home/	165	48.2
	http://www.dadgroup.com/	52	15.2
	http://www.joswe.com/	35	10.3
	Others	90	26.3
Total		342	100
Gender	Male	124	36.3
	Female	218	63.7
Total		342	100
Age	Below 20 years	34	9.9
	21–25 years	127	37.2
	26–30 years	77	22.5
	31–35 years	54	15.8
	36–39 years	27	7.9
	Above 40 years	23	6.7
Total		342	100
Highest level of education	Diploma	39	11.4
	Bachelor's degree	262	76.6
	High diploma	25	7.3
	Masters or higher	16	4.7
Total		342	100
Status	Student only	111	32.5
	Employee only	38	11.1
	Both	193	56.4
Total		342	100

### Measurement model

The evaluation of the reflective signs in the measurement model in the SmartPLS depends on four factors: the discriminant validity, convergent validity, individual item reliability, and construct reliability (100). The reliability of the individual items was considered sufficient in this research because the loadings of all the dimensions and indicators exceeded 0.714 (Table 4).

**Table 4:** Model assessment.

**Dimension/construct/indicator**	**Items**	**Loading**	**Cronbach's alpha**	**Composite reliability**	**Average variance extracted (AVE)**
Enduring involvement (*EI*)	EI1	0.797	0.827	0.880	0.597
	EI2	0.816			
	EI3	0.855			
	EI4	0.714			
	EI5	0.759			
Perceived risk (*PR*)	PR1	0.853	0.864	0.905	0.705
	PR2	0.736			
	PR3	0.874			
	PR4	0.887			
Resistance to change (*RC*)	RC1	0.879	0.838	0.902	0.755
	RC2	0.883			
	RC3	0.843			
Product description (*PD*)	PD1	0.791	0.688	0.827	0.615
	PD2	0.799			
	PD3	0.762			
Product awareness (*PA*)	PA1	0.853	0.814	0.890	0.729
	PA2	0.859			
	PA3	0.850			
Intention to use MMSEOA (*ITU*)	ITU1	0.869	0.891	0.924	0.753
	ITU2	0.857			
	ITU3	0.883			
	ITU4	0.861			

All the variables were multidimensional, and their dimensions match the requisite of the composite reliabilities; their construct reliability exceeded 0.7 (Table 4). To evaluate the convergent validity, the average variance extracted (AVE) was examined. In this case, all the latent constructs achieved convergent validity, as the corresponding AVE crossed the 0.5 level (Table 4). Finally, Table 5 reveals that all the variables achieved discriminant validity, according to the Fornell–Larcker criterion (103, 104). This indicates that all the constructs were empirically distinct.

**Table 5:** Measurement of model discriminant validity.

	**ITU**	**EI**	**PR**	**PA**	**PD**	**RC**
ITU	0.868					
EI	0.537	0.773				
PR	0.141	0.055	0.840			
PA	0.597	0.468	0.237	0.854		
PD	0.305	0.241	0.149	0.392	0.784	
RC	0.512	0.403	0.135	0.560	0.260	0.869

EI, enduring involvement; PR, perceived risk; RC, resistance to change; PD, product description; PA, product awareness; ITU, intention to use MMSEOA. Fornell–Larcker criterion: Diagonal elements (bold) are the variance square root of shared among the variables and their measures (AVE). Off-diagonal elements are associations among variables. For validity of discriminant, diagonal elements must be greater than off-diagonal elements.

### Structural model

**Figure 2:**
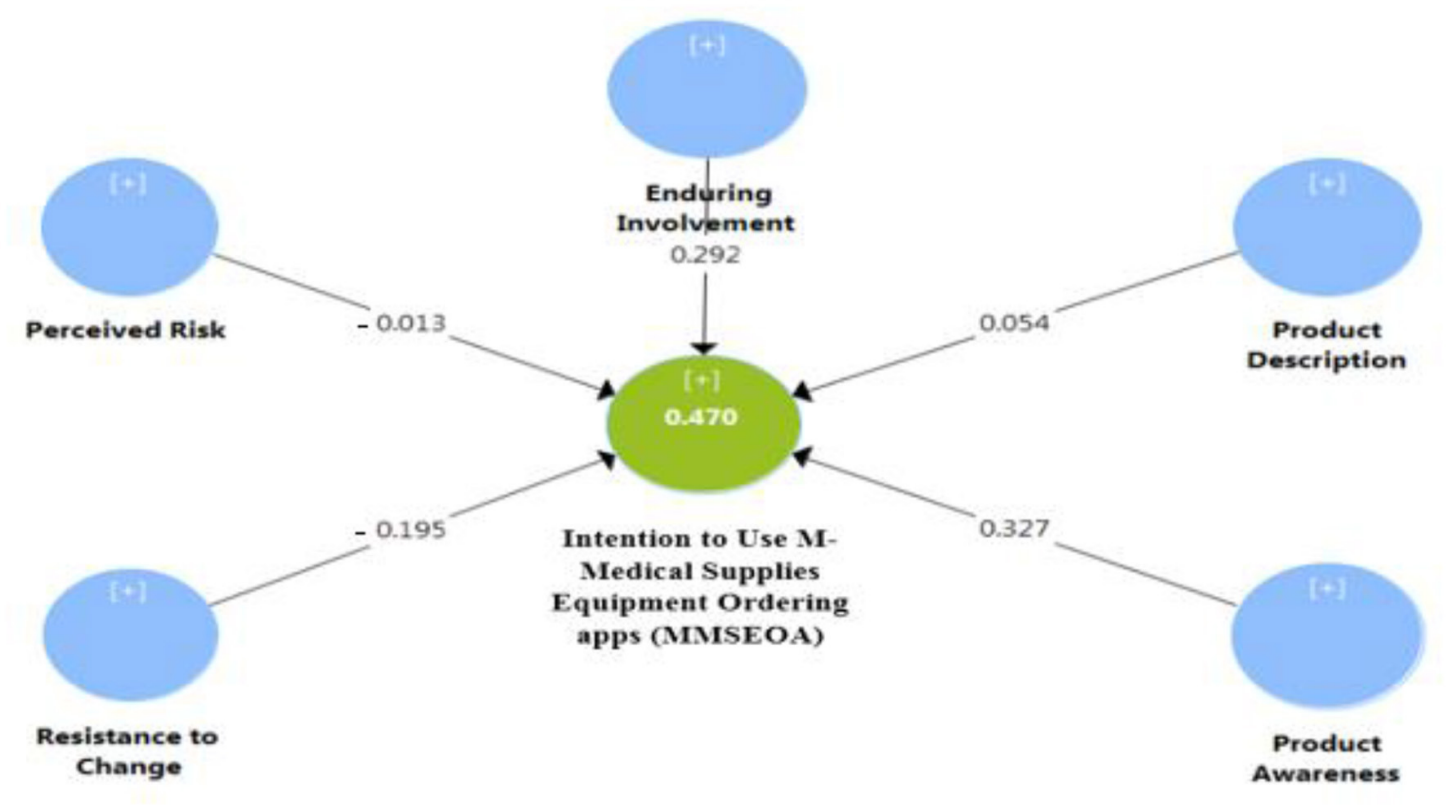
Model path findings.

**Figure 3:**
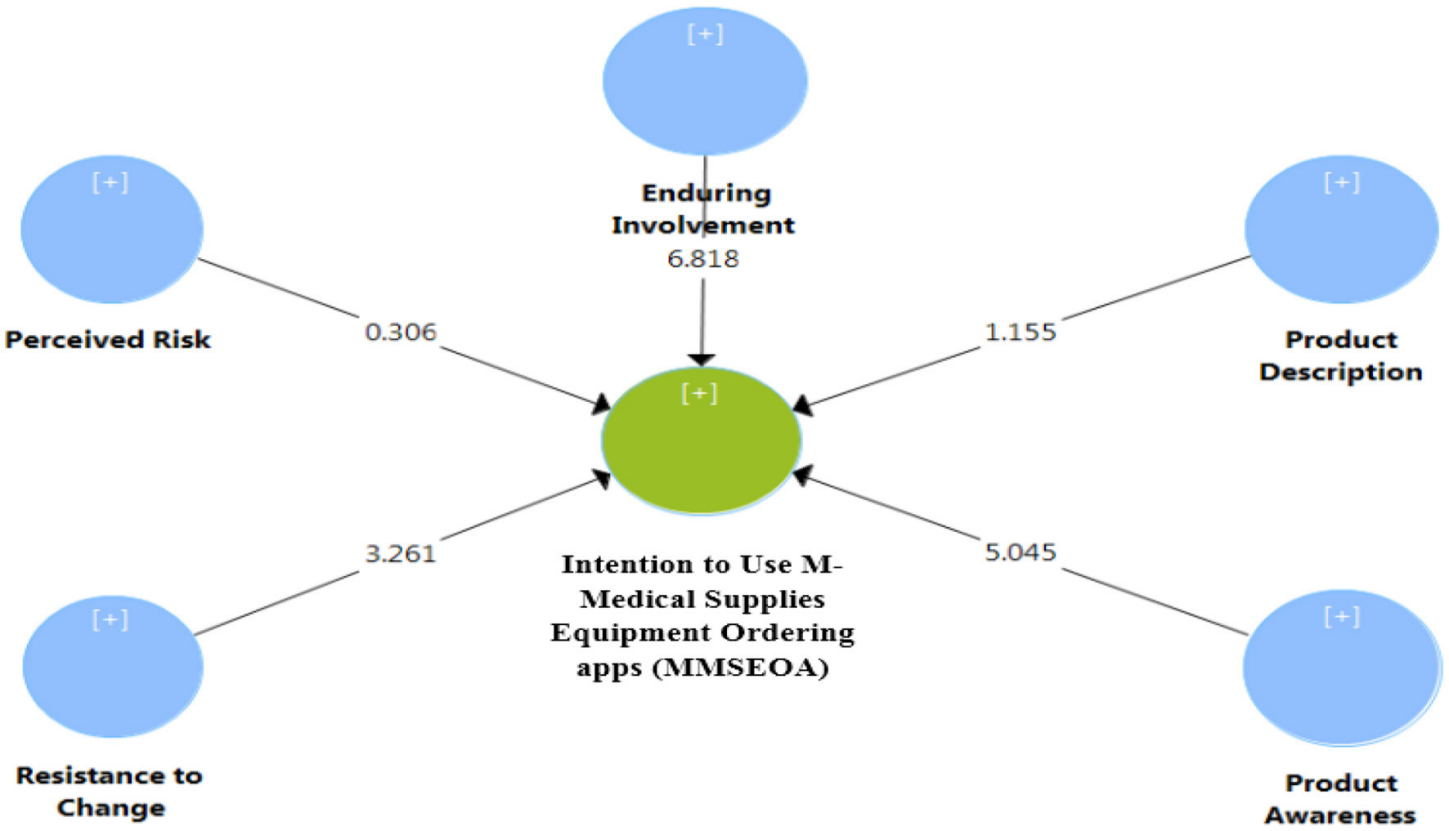
Model path importance findings.

All the identified variables enabled the evaluation of the structural model. The significance of the structural path coefficients (Figures 2, 3), R^2^ and Q^2^ were reported. Similar to (103), t-statistics and confidence intervals were created using bootstrapping (500 resamples). This enabled the researchers to statistically evaluate the importance of the path coefficients. As shown in Table 6 below, three out of the five direct impacts, H1, H3, and H5, were significant and supported. H2 and H4 were not supported. The hypothesized relationships were examined. Further, the structural framework was tested, and the outcomes were summarized and described in detail in Tables 6, 7.

**Table 6:** Inner structural model results.

**Hypotheses**	**Original sample**	**Sample**	**Standard**	***T*-values**	***P*-values**	**Decision**
**relationship**	**(Std.Beta)**	**mean (M)**	**error (STERR)**	
Enduring Involvement -> (MMSEOA) 7	0.292	0.289	0.043	6.818	0.000***	Supported
Perceived Risk -> (MMSEOA)	−0.013	0.018	0.044	0.306	0.760	Not Supported
Resistance to Change -> (MMSEOA)	−0.195	0.201	0.060	3.261	0.001***	Supported
Product Description -> (MMSEOA)	0.054	0.057	0.047	1.155	0.249	Not Supported
Product Awareness -> (MMSEOA)	0.327	0.324	0.065	5.045	0.000***	Supported

Significant at *p < 0.1; **p < 0.05; ***p < 0.01.

**Table 7:** Hypotheses overview.

**Hypothesis**	**Hypothesized path**	**Decision**
*H1*	Enduring involvement is positively linked to the intention of using MMSEOA.	Supported
*H2a*	Product description is positively linked to the intention of using MMSEOA.	Not supported
*H2b*	Product awareness is positively linked to the intention of using MMSEOA.	Supported
*H3a*	Perceived risk is negatively linked to the intention of using MMSEOA.	Not supported
*H3b*	Resistance to change is negatively linked to the intention of using MMSEOA.	Supported

The study framework appeared to possess a suitable predictive power for dependent variables, as explained by Tables 6, 8. Hence, the intention to use MMSEOA achieved the highest explained variance (*R*^2^ = 0.470) in Table 8. Furthermore, the researchers used a cross-validated redundancy index (*Q*^2^) to assess the model of the endogenous reflective variables. As shown in Table 8, the *Q*^2^ was larger than 0, which indicates that the framework has predictive relevance. As shown in Table 8, the result proves that the structural model has adequate predictive relevance for the endogenous construction of the intention to use MMSEOA.

**Table 8:** Predictive quality of framework.

**Construct**	**Type of**	***R* ^2^**	**Redundancy**	**Communality**	**Result**
	**variable**		**of cross-**	**of cross-**	
	**variable**		**validity**	**validity**	
MMSEOA	Endogenous	0.470	0.345	0.574	Moderate

Moreover, the independent variables (enduring involvement, resistance to change, and product awareness), with the exception of the perceived risk and product description variables as an interpretive construct of intention to use the MMSEOA, embody key antecedent constructs and significantly impact the dependent variables. After examining the value of *f*^2^ (105, 106), *f*^2^ has to be more than the base level of 0.02, as shown in Table 9 (100). For three of the constructs (enduring involvement, resistance to change, and product awareness), *f*^2^ was more than 0.02; however, for the other two (perceived risk and product description), *f*^2^ was less than the threshold value. Finally, the researcher calculated the goodness of fit (GoF) of our model as the root of the result of *R*^2^^*^ the AVEs for all the constructs (107). The GoF of the proposed framework was 0.570, higher than 0.36, which indicates a large GoF (107). It was calculated using the following formula:


$$GoF=\sqrt{\left(0.470{\;}^{*}0.692\right)}=\;0.570$$

**Table 9:** Effect size of exogenous constructs.

**Endogenous**	**Exogenous**	**Effect size**	**Result**
**construct**	**constructs**	
MMSEOA	Enduring involvement	0.120	Small effect
	Perceived risk	0.000	No effect
	Resistance to change	0.111	Small effect
	Product description	0.005	No effect
	Product awareness	0.048	Small effect

## Discussion and implications

### Discussion

This research was conducted on the usage of the MMSEOA model at different levels of refinement, along with the consideration of the reasons for enduring involvement, product description and product awareness and reason against including perceived risk and resistance to change. The results of this study revealed that three (enduring involvement, resistance to change, and product awareness) of the five constructs identified significantly impacted the intention to use the MMSEOA. However, the other two constructs (perceived risk and product description) had no clear effect on the MMSEOA.

The foremost theoretical enhancement achieved by the current research is the creation of a model based on BRA, as shown in Figure 1. This framework represents the two stages (i.e., reasons for and reasons against) that customers (clinic doctors) move through in creating the process of purchase intention on MMSEOAs. The experimental findings reinforced the validity of the research framework. Based on the above framework, the researcher discovered that customers (clinic doctors) on MMSEOAs shifted with time through the succeeding tracks of reasons for including enduring involvement (platform emotion), product description, product awareness (familiarity with the product), reasons against, namely perceived risk, resistance to change (fear effect), and intention to use the MMSEOA.

Contrary to expectations, the empirical analysis revealed that both the perceived risk had no significant impact on behavioral intentions. For the perceived risk, as is known, younger consumers are less risk-averse than older individuals (108). Most of the respondents in the current study were clinic doctors between 21 and 25 years old (37.2%), and 26 and 30 years (22.5%). Both age groups comprised 59.7% of the total sample, as mentioned in Table 3. It is expected that their lower perceived risk was related to their age. Similarly, the influence of the perceived risk on the behavioral intention in the current study may be minimal because of the age of the respondents (93). This research also revealed that product descriptions had no influence on the behavioral intention of clinic doctors because they were very knowledgeable on medical supplies equipment details. Therefore, based on the findings, this is principally responsible for their indifference toward a construct of the product description.

### Theoretical implications

This research has a considerable contribution to the body of knowledge on adopting MMSEOA. The current study has three key theoretical contributions. First, the study's results demonstrate a more comprehensive understanding of the relative impact of “reasons for” and “reasons against” in influencing the customers' intention to use MMSEOA. This was important because most previous research considered the determinants affecting the adoption of MMSEOA. In comparison, there was little research on understanding the potential barriers that prevented individuals from using MMSEOA. Second, this is the first empirical study to use BRA to investigate the adoption of MMSEOA. This study explains the MMSEOA adoption behavior of doctors in Jordon. However, the existing research significantly contributes to the MMSEOA's research stream. Third, the results offer significant insights into Jordan customers' behavior and beliefs about their propensity to use the MMSEOA. Still, there is currently a lack of knowledge regarding the perceptions of Jordan customers toward using MMSEOA. These results can persuade other researchers to perform similar research in various geographical and cultural groups to enhance their knowledge on this topic.

### Practical implications

Based on the results, it is recommended that the services, products, and main function of MMSEOAs be researched further to reveal their possible influences on customers' intention to use. Unlike traditional e-commerce, customers on MMSEOA (clinic doctors), retailers (pharmaceutical companies, or even drug stores), and platform providers are all new to the MMSEOA environment. Consequently, BRA may help researchers recognize novel implications and insights in that context.

In addition, several practical implications are recommended from the research outcomes, especially for product providers who execute marketing plans on MMSEOA. Furthermore, service providers must pay more attention to the creation of greater product awareness, which can be achieved *via* modes of marketing that improve customers' familiarity with the product, than to the description of the goods sold on their MMSEOA. Globally, providers must offer sufficient, clear, and concise information on their goods and attract customers' attention through different methods, such as utilizing images or even videos. This will increase customers' knowledge of MMSEOA products and enhance purchasing attitudes and intention to buy.

Furthermore, in view of the characteristics and functions afforded by MMSEOA, it is necessary to understand the purchasing practices of customers in various regions and countries. Therefore, product providers must formulate a new purchasing environment that is favorable to customers to complete the purchase. Product providers must also satisfy the needs of potential customers' needs, respond to requests for information, and encourage customers to spend as much time as possible browsing the MMSEOA, beyond just making actual purchases. Product providers can further develop a connection with their customers by enlightening them on how to find products, browse through the app, efficiently utilize its main functions, and appropriately buy services or products. The aforementioned will possibly raise customers' engagement with MMSEOAs, which can improve buying habits.

### Limitations and future directions of the study

The present study has a few limitations that future researchers can address. First, the results are not generalizable to medical supply order app users because only clinic doctors and their secretaries were considered. Second, because of various resources and time constraints, a sample of only 342 subjects (clinic doctors and secretaries) was studied, which greatly limits our ability to generalize our findings to these other apps. In future, research may be extended to include hospital doctors and marketers of medical supplies, to generalize the findings of this study. This will be helpful in providing more extensive and deeper outcomes. Additionally, this study explained only the effective response routes that affected customers' intention to use MMSEO. Third, the current research has considered the “reasons for and against” to study the customers' intention to use MMSEO. Future researchers can use the complete behavioral reasoning approach by including all the block elements of BRA: values, reasons for, reasons against, attitude and intention to use. In addition, there is gap between the customers' intention and actual behavior. The future researchers can examine the actual behavior of the customers.

## Conclusion

In the context of traditional e-commerce, familiarity with goods was not regarded as the main factor influencing consumers' behavior intention. In this study, reasons for (i.e., the platform emotion stage, familiarity with products) significantly and positively influence the customers' intention to use the MMSEOA but product description had no significant influence on intention to use the MMSEOA. On the other hand, reasons against resistance to change were examined and found to positively influence the intention to use the MMSEOA. However, the results showed that the perceived risk had no effect on the intention to use the app. Further, the impact of product awareness and description, in relation to the differences in the cultural background of the consumer and linguistic differences, should be considered. Moreover, establishing familiarity with a product requires navigating a process to impact the intention to use the MMSEOA. The researchers are convinced of the necessity of considering the impacts of good classification or recognition and examining such impacts on intention to use MMSEOA. Whereas, previous studies examined only the situational involvement and its influence on purchase intention.

## Data availability statement

The original contributions presented in the study are included in the article/supplementary material, further inquiries can be directed to the corresponding author.

## Author contributions

The author confirms being the sole contributor of this work and has approved it for publication.

## Conflict of interest

The author declares that the research was conducted in the absence of any commercial or financial relationships that could be construed as a potential conflict of interest.

## Publisher's note

All claims expressed in this article are solely those of the authors and do not necessarily represent those of their affiliated organizations, or those of the publisher, the editors and the reviewers. Any product that may be evaluated in this article, or claim that may be made by its manufacturer, is not guaranteed or endorsed by the publisher.
